# Preoperative Magnetic Resonance Imaging Radiomics for Predicting Early Recurrence of Glioblastoma

**DOI:** 10.3389/fonc.2021.769188

**Published:** 2021-10-27

**Authors:** Jing Wang, Xiaoping Yi, Yan Fu, Peipei Pang, Huihuang Deng, Haiyun Tang, Zaide Han, Haiping Li, Jilin Nie, Guanghui Gong, Zhongliang Hu, Zeming Tan, Bihong T. Chen

**Affiliations:** ^1^ Department of Radiology, Xiangya Hospital, Central South University, Changsha, China; ^2^ National Clinical Research Center for Geriatric Disorders, Changsha, China; ^3^ Hunan Engineering Research Center of Skin Health and Disease, Changsha, China; ^4^ Hunan Key Laboratory of Skin Cancer and Psoriasis, Changsha, China; ^5^ National Clinical Research Center for Geriatric Disorders (Xiangya Hospital), Central South University, Changsha, China; ^6^ Department of Pharmaceuticals Diagnosis, GE Healthcare, Hangzhou, China; ^7^ Department of Pathology, Xiangya Hospital, Central South University, Changsha, China; ^8^ Department of Neurosurgery, Xiangya Hospital, Central South University, Changsha, China; ^9^ Department of Diagnostic Radiology, City of Hope National Medical Center, Duarte, CA, United States

**Keywords:** blood urea nitrogen, glioblastoma, magnetic resonance imaging, nomogram, preoperative, radiomics, recurrence, Visually Accessible Rembrandt Images (VASARI)

## Abstract

**Purpose:**

Early recurrence of glioblastoma after standard treatment makes patient care challenging. This study aimed to assess preoperative magnetic resonance imaging (MRI) radiomics for predicting early recurrence of glioblastoma.

**Patients and Methods:**

A total of 122 patients (training cohort: n = 86; validation cohort: n = 36) with pathologically confirmed glioblastoma were included in this retrospective study. Preoperative brain MRI images were analyzed for both radiomics and the Visually Accessible Rembrandt Image (VASARI) features of glioblastoma. Models incorporating MRI radiomics, the VASARI parameters, and clinical variables were developed and presented in a nomogram. Performance was assessed based on calibration, discrimination, and clinical usefulness.

**Results:**

The nomogram consisting of the radiomic signatures, the VASARI parameters, and blood urea nitrogen (BUN) values showed good discrimination between the patients with early recurrence and those with later recurrence, with an area under the curve of 0.85 (95% CI, 0.77-0.94) in the training cohort and 0.84 [95% CI, 0.71-0.97] in the validation cohort. Decision curve analysis demonstrated favorable clinical application of the nomogram.

**Conclusion:**

This study showed the potential usefulness of preoperative brain MRI radiomics in predicting the early recurrence of glioblastoma, which should be helpful in personalized management of glioblastoma.

## Introduction

Glioblastoma multiforme (GBM) is the most malignant primary brain tumor (1) and represents one third of primary brain tumors with 79,000 new cases worldwide per year (2). The standard treatment for newly diagnosed GBM is maximal surgical resection followed by radiotherapy plus temozolomide (3), which takes about six months (4–6). Nevertheless, early recurrence may occur due to the aggressiveness and diffuse infiltrative growth of GBM (7, 8).

Pretreatment identification of patients at risk for early GBM recurrence has several benefits (9). First, a more aggressive treatment strategy, such as a more extensive resection (10), with concurrent extended individualized radiotherapy or new radiotherapy-based methods, may be warranted. Second, patients may need to choose new treatment early in the course of the disease to avoid delays in treatment (11, 12). Finally, patients with early occurrence may need additional in-depth testing, such as gene sequencing, to assist in clinical decision-making. Previous studies have identified various indicators associated with risk of GBM recurrence such as age, gender, Karnofsky performance status (KPS), and pathology findings (13–17), but no reliable tool is available to accurately predict early recurrence. Molecular pathology and genotyping may be helpful in assessing recurrence (18, 19). However, tissue collection is invasive and may lead to misdiagnosis due to errors in the sampling of heterogenous tumors. Clinicians typically use brain MRI to evaluate the visual radiological features of GBM such as the size, location, edema and enhancement characteristics. However, conventional MRI evaluation is not adequate for predicting early recurrence in GBM (19, 20). Therefore, there is a need to assess additional imaging biomarkers analyzed by computational methods for predicting GBM recurrence (21, 22).

Radiomics quantifies high-dimensional imaging features of tumors (23, 24), which may reflect tumor heterogeneity and molecular pathology. Radiomics has been used to evaluate GBM recurrence because a large number of image features can be extracted from brain MRI data, including the contrast enhanced sequence, fluid attenuation inversion recovery (FLAIR), T1-weighted imaging, and T2-weighted imaging (23–26). In addition, GBM is a highly vascularized tumor, and the extent of vascularization is directly associated with the prognosis and recurrence for patients with GBM (24, 25, 27, 28). Hence, it is reasonable to speculate that MRI radiomics, taking into consideration of the various imaging features including enhancing characteristics reflecting tumor vascularity, could be a potentially useful tool to predict early recurrence of GBM.

In this study, we evaluated preoperative brain MRI scans for both radiomic features analyzed by computational methods and the conventional radiological characteristics included in the Visually Accessible Rembrandt Images (VASARI) feature set assessed by neuroradiologists through visual inspection (6). We built a nomogram incorporating radiomic features, clinical variables, and the VASARI parameters to predict early recurrence of GBM. We hypothesized that integration of conventional radiological parameters and clinical variables into the computational radiomic model could improve the model performance for differentiating patients with and without early recurrence of GBM

## Patients and Methods

### Patients

Ethical approval was obtained from the Ethics Committee and Institutional Review Board of Xiangya Hospital of Central South University, P. R. China (IRB number: 201607831). Informed consent was waived due to the retrospective nature of the study.

**Figure 1:**
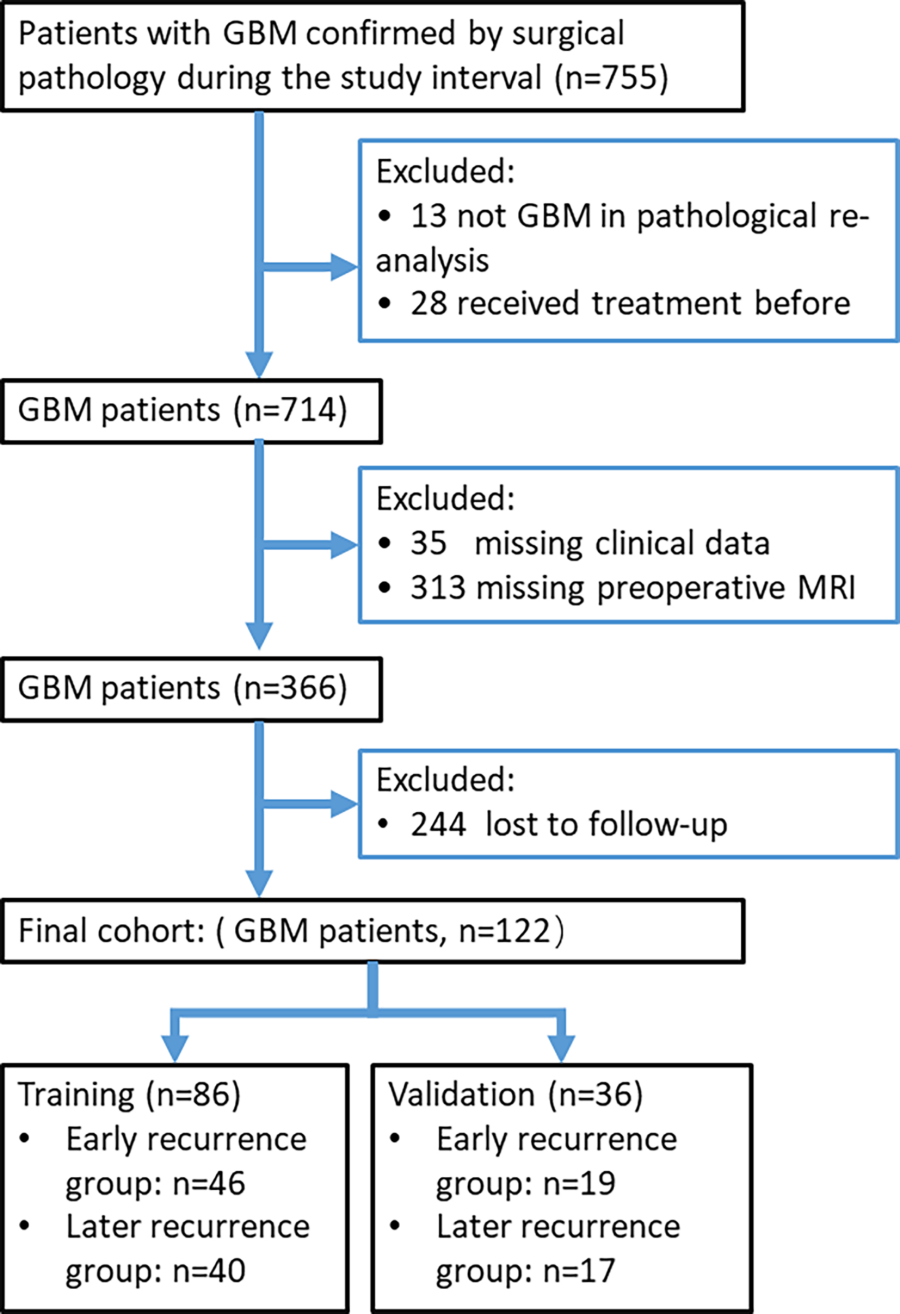
Study recruitment process for patients with glioblastoma multiforme (GBM).

The study cohort consisted of all patients between July 2010 and April 2018 who had GBM that was pathologically confirmed from surgical specimens. The patients were identified by searching our institutional data base and medical chart review. We included only the patients with preoperative brain MRI scans obtained less than 14 days prior to surgery. Patients with missing clinical data and those who received treatment before surgery, such as radiotherapy, chemotherapy, or chemoradiotherapy, were excluded. Patients with a progression-free survival (PFS) less than 7 months were assigned to the early recurrence group (7, 8), and the remaining patients were assigned to the later recurrence group. **Figure 1** presents a study flow diagram.

### Re-Assessment of Pathology Results

All pathology slides for the study cohort were re-assessed by two pathologists specializing in brain tumors to confirm GBM diagnosis (G.G. and Z.H., with 10 and 25 years of experience, respectively). Both pathologists were blinded to the clinicopathological data. If any discrepancies arose, agreements were reached by consensus.

### Tumor Segmentation and Radiomic Feature Extraction

Brain MRI imaging was performed on a 3T MRI scanner (Discovery MR750w, GE Healthcare) or a 1.5T MRI scanner (MAGNETOM Avanto, Siemens Healthineers, Erlangen, Germany). All images were retrieved from the Picture Archiving and Communication System (PACS) at our hospital. All brain MRI scans were reviewed independently by two neuroradiologists [reader 1 (H.D.) and reader 2 (Z.H.), with 5 and 25 years of experience, respectively] who were blinded to the patient information. Any disagreements were resolved in a panel format with two additional researchers (X.Y. and H.T.).

All conventional radiological findings from the brain MRI images, as assessed by the neuroradiologists visually, were evaluated according to the VASARI parameters *(* https://wiki.nci.nih.gov/display/CIP/VASARI *)*. There were 29 VASARI parameters recorded as F1-F29 (6).

**Figure 2:**
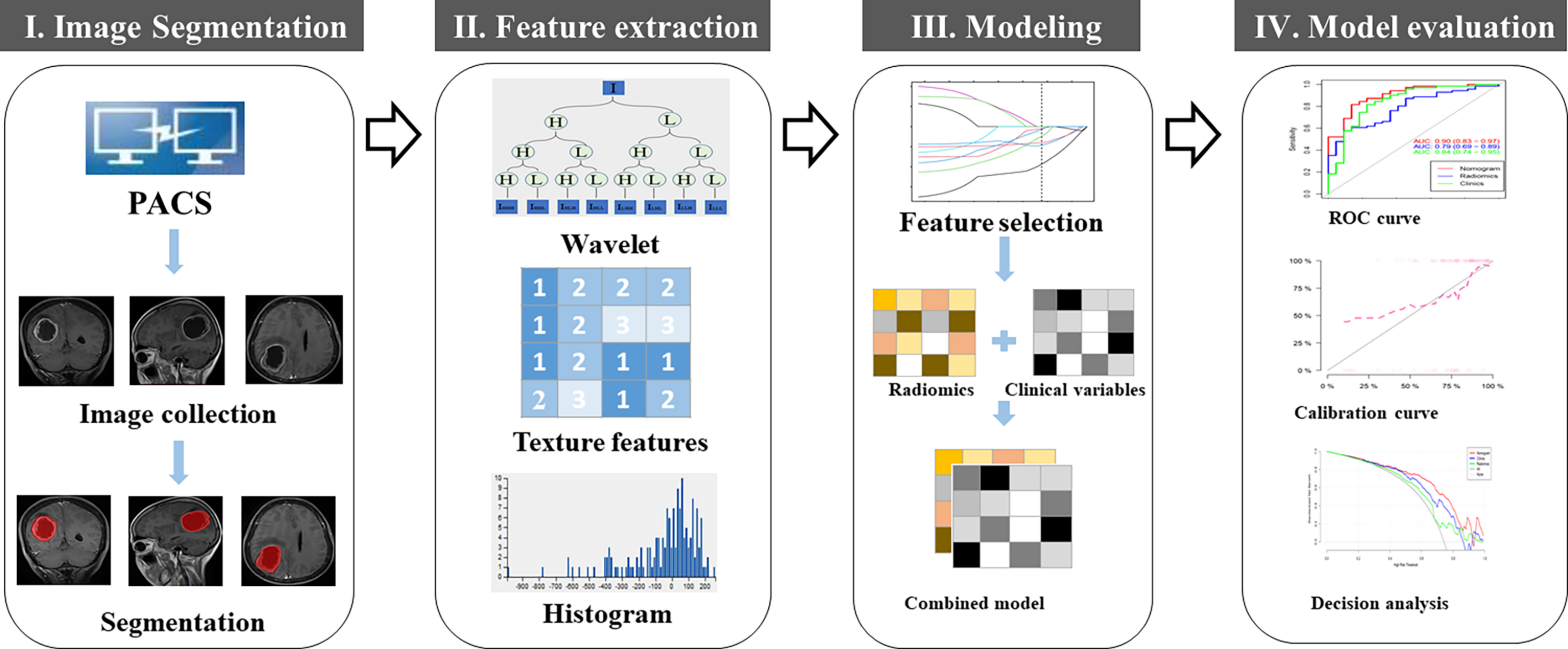
Workflow for tumor segmentation, radiomic feature extraction and predictive modeling.

Image pre-processing, tumor segmentation, and radiomic feature extraction were performed as described previously (29). Wavelet transform and Laplacian of Gaussian (LoG) filtering were used for image denoising. For each GBM tumor, manual contouring and segmentation were performed on the axial, sagittal, coronal images of the tumor at the level with the largest tumor dimension, as determined by both neuroradiologists (readers 1 and 2) who defined the margins of the tumors. The segmented tumor with regions of interest (ROI) was saved for subsequent radiomic feature extraction. We extracted reliable features from the original image and its corresponding filtered image. A total of 1204 quantitative radiomic features were extracted from each MRI image including first order statistics, shape, gray-level run-length matrix (GLRLM), gray-level co-occurrence matrix (GCLM), neighboring gray tone difference matrix (NGTDM), gray level dependence matrix (GLDM), and gray-level size zone matrix (GLSZM), using an open-source python package PyRadiomics (2.2.0) (http://www.radiomics.io/pyradiomics.html *)* (30). To remove the potential differences between MRI images acquired from the two different MRI scanners, normalization with the final 256 bins was performed on all original MRI images using the gray-scale discretization method before extracting the radiomic features (Analysis Kit software, version V3.0.0.R, GE Healthcare). **Figure 2** presents the workflow for tumor segmentation, radiomic feature extraction and selection, and predictive modeling.

### Radiomic Feature Selection, Predictive Modeling and Statistical Analysis

Max-Relevance and Min-Redundancy (mRMR) was performed to eliminate the redundant and irrelevant features (31), and 20 features were retained. Then, the least absolute shrinkage and selection operator (LASSO) method was used to select the optimized subset of features to construct the radiomic signature (Rad-score) and to build the models. If the Pearson correlation coefficient of the feature pairs was larger than 0.8, one of them was deleted. A 10-fold cross-validation was conducted to avoid model overfitting. The optimal parameter λ was obtained in terms of the largest value of lambda so that the error was within 1 standard error of the minimum. According to λ, the features corresponding to the non-zero LASSO coefficient were selected (see **Supplementary Figure 1S**). Multivariate logistic regression analysis was applied to develop a model for predicting early recurrence of GBM and the nomogram was generated (**Figure 2**).

Statistical analysis was conducted with R software (version 3.0.1; http://www.Rproject.org). The reported statistical significance levels were all two-sided, with statistical significance set at 0.05.

### Model Performance and the Nomogram

Calibration curves (Hosmer-Lemeshow H test) were used to evaluate the calibration of the models, and receiver-operating characteristic (ROC) curves were performed to access the differentiation efficiency. The performance of the internally validated nomogram was tested in the validation cohort. The logistic regression formula formed in the primary cohort was applied to all patients of the validation cohort. Then, the calibration curve was derived on the basis of the regression analysis, and the clinical usefulness of the models was evaluated by using decision curve analysis in the validation cohort. Decision curve analysis was conducted to determine the clinical usefulness of the radiomic nomogram by quantifying the net benefits at different threshold probabilities in the validation dataset. Additionally, a correlation matrix analysis was performed to evaluate the correlations among all selected features that were integrated into the final predictive model.

## Results

### Patient Information

A total of 122 patients with GBM were included in this study, including 65 with early recurrence. Median PFS for all patients was 189 ± 19 (range, 26 -1639) days after surgery, with the PFS for early recurrence group and later recurrence group being 97 ± 8 (range, 26 - 203) and 326 ± 31 (range, 217 - 1639) days, respectively. Each patient was randomly assigned to the training cohort (n=86) or the validation cohort (n=36) at a ratio of 5:2. Patient information and comparison between the training and validation cohorts are summarized in **Table 1**. No variables were statistically different between the training and the validation cohorts (P > 0.05), ensuring a reasonable classification.

**Table 1:** Demographic, clinical, laboratory, VASARI parameters and radiomic score (Rad-score) of the 122 patients with glioblastoma (GBM).

Characteristic	Total (n=122)	Early recurrence (n=65)	Later recurrence (n=57)	*P*-Value	Training cohort (n=86)	Validation cohort (n=36)	P*-*Value
Demographics and clinical characteristics							
Gender, n (%)							
Male	72 (59.0)	35 (53.8)	37	0.218	49 (57.0)	23 (63.9)	0.613
Female	50 (41.0)	30 (46.2)	20		37 (43.0)	13 (36.1)	
Age [median (IQR), years]	47 (34~55)	47.0 (34.5~57.0)	47.0 (34.0~54.5)	0.683	47.5 (32~57)	47.0 (34.3~52.5)	0.678
KPS [mean (SD)]	81.2 (13.4)	80.15 (13.75)	82.5 (13)	0.346	80.2 (15.3)	83.6 (6.8)	0.203
Laboratory findings							
BUN [No. (%)]							0.367
Elevated	23 (18.9)	18 (27.7)	46 (80.7)	0.175	59 (68.6)	28 (77.8)	
Normal	87 (71.3)	41 (63.1)	5 (8.8)		19 (22.1)	4 (11.1)	
Decreased	12 (9.8)	6 (9.2)	6 (10.5)		8 (9.3)	4 (11.1)	
VASARI parameters [mean (SD)]							
F5	4.467 (1.03)	4.6 (1.028)	4.316 (1.02)	0.038*	4.4 (1.1)	4.6 (0.9)	0.421
F21	1.26 (0.44)	1.38 (0.49)	1.12 (0.33)	0.002**	1.29 (0.46)	1.17 (0.38)	0.144
F26	7.79 (0.55)	7.68 (0.64)	7.91 (0.39)	0.007**	7.77 (0.57)	7.83 (0.51)	0.477
Radiomics							
Rad-score (median [interquartile range])	0.4[-0.4,1.4]	1[0.2,1.6]	-0.3 [-1.4, 0.4]	<0.001***	0.4 [-0.8, 1.2]	0.9 [-0.5, 2.0]	0.176

*P<0.05, **P<0.01 and ***P<0.001.

BUN, Blood urea nitrogen; KPS, Karnofsky performance status; VASARI, Visually Accessible Rembrandt Images; F5, the VASARI parameter indicating enhancing tumor; F21, the VASARI parameter indicating deep white matter invasion; F26, the VASARI parameter indicating extent of tumor resection.

### Inter-Observer and Intra-Observer Reproducibility of Radiomic Features

Satisfactory inter- and intra-observer reproducibility of the texture feature extraction was achieved. The inter-observer intraclass correlation coefficients (ICCs) calculated based on features extracted by reader 1 (first extraction) and reader 2 ranged from 0.791 to 0.897. The intra-observer ICCs, calculated based on reader 1’s feature extraction results from two attempts, ranged from 0.804 to 0.901. Therefore, all subsequent analyses were based on the radiomic features extracted by reader 1.

### Predictive Model Building and Model Performance

Of all the radiomic features, 20 potential predictors were retained based on data from the 86 patients in the training cohort. Features with nonzero coefficients in the LASSO logistic regression model were used (**Supplementary Figure 1S**). The most predictive subset of features was selected and the corresponding coefficients were evaluated (**Supplementary Figure 2S**).

**Figure 3:**
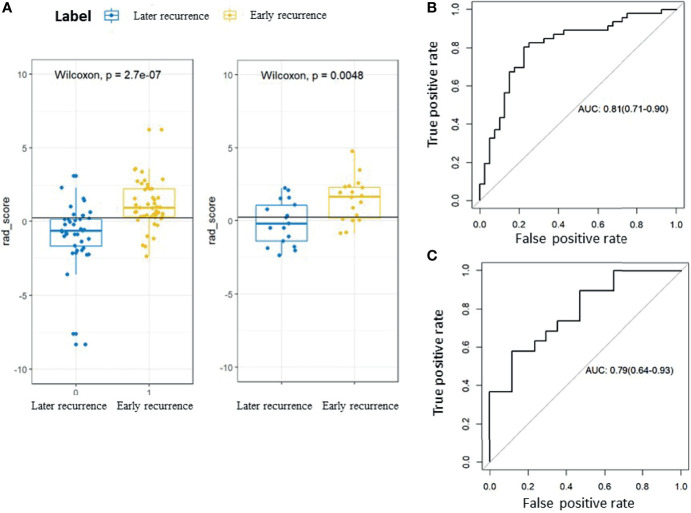
Rad-scores and receiver-operating characteristic (ROC) curves for the early recurrence group and the later recurrence group. **(A)** Box plots showing the Rad-scores for the early recurrence group and the later recurrence group. The label 0 indicates the later recurrence group and 1 indicates the early recurrence group. The left panel shows the training cohort and the right panel shows the validation cohort. **(B)** ROC curve for the training cohort. **(C)** ROC curve for the validation cohort.

A significant increase in the Rad-scores for the early recurrence group compared to the later recurrence group was found in the training cohort (P < 0.001), which was further confirmed in the validation cohort (P < 0.005). The radiomic signature yielded an area under the curve (AUC) of 0.81 (95%CI 0.71–0.90) in the training cohort and 0.79 (95%CI 0.64–0.93) in the validation cohort. The ROC curves are presented in **Figure 3**.

Regarding the conventional radiological findings according to the VASARI parameters, we found that F5 for tumor enhancement (Odds Ratio=1.61) and F21 for deep white matter invasion (Odds Ratio=3.45) were risk factors for predicting early recurrence, and F26 indicating the extent of tumor resection (Odds Ratio=0.51) was a protective factor against early recurrence. In our study, F5 and F21 were predictors of early recurrence, and F26 predicted the lack of early recurrence.

**Figure 4:**
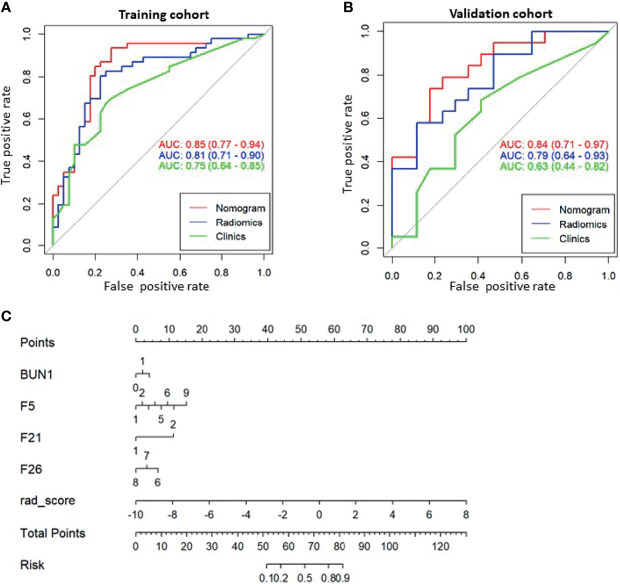
The receiver operating characteristic (ROC) curves of the predictive models and the corresponding nomogram. ROC curve for the combined model (Nomogram, red), radiomic model (Radiomics, blue), and clinical model (Clinics, green) for **(A)** the training cohort and **(B)** the validation cohort. **(C)** Nomogram with significant indicators. F5, F21, and F26 are part of the Visually Accessible Rembrandt Image (VASARI) feature set. F5, enhancing tumor; F21, deep white matter invasion; F26, extent of tumor resection.

Based on the radiomic signature and clinical variables, a model was constructed to predict early recurrence. A logistic regression analysis identified the radiomic signature (F5, F21, F26) and blood urea nitrogen (BUN) concentration as independent predictors (**Figures 4A, B**), which was presented as a nomogram (**Figure 4C**). The model with the nomogram provided an AUC of 0.85 (0.77-0.94) in the training cohort and 0.84 (0.71-0.97) in the validation cohort (**Figures 4A, B**). The calibration curve for the probability of resistance in the training and validation cohorts showed good agreement between prediction and observation.

### Clinical Applications

**Figure 5:**
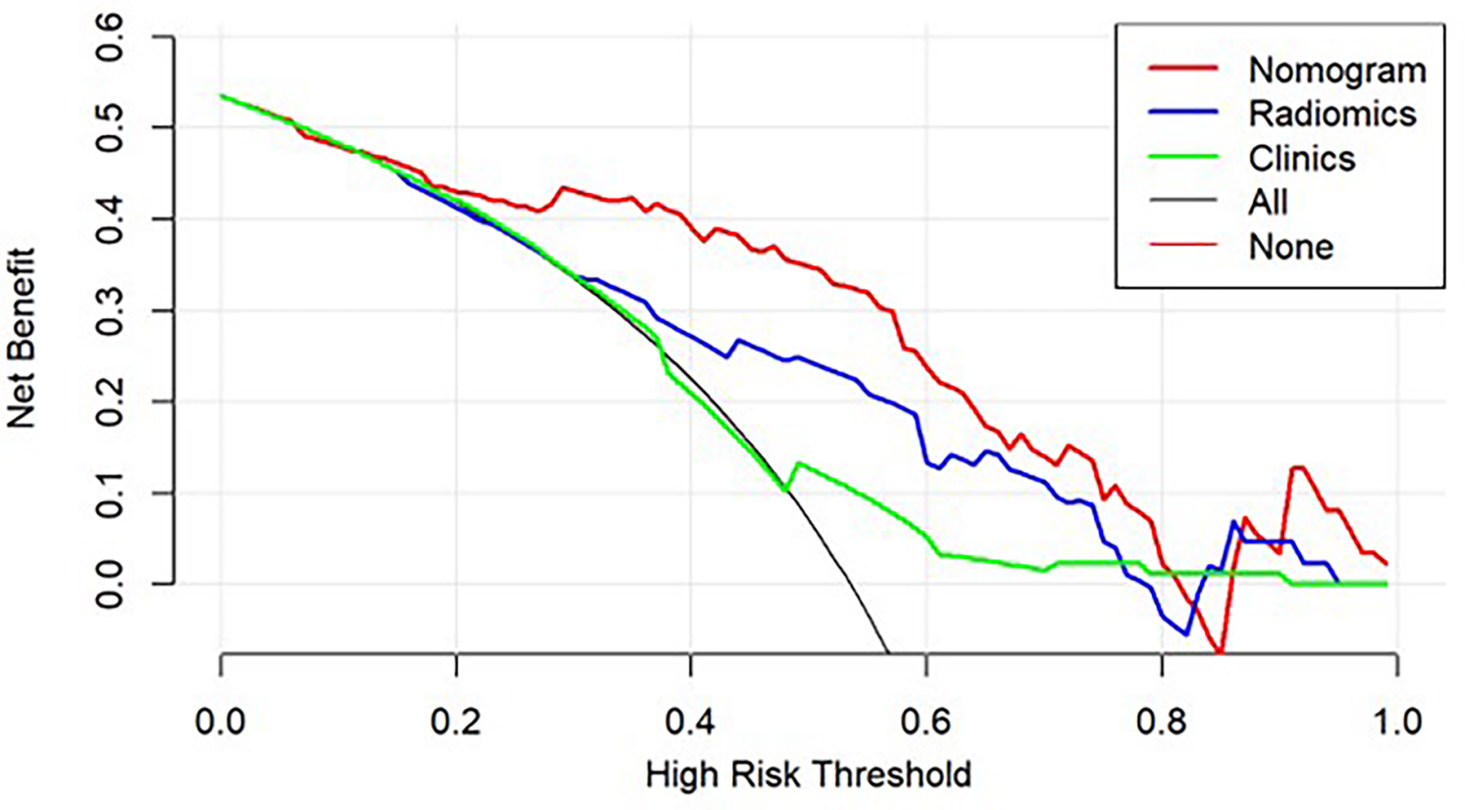
Decision curve analysis for the models built with the nomogram (red), radiomic model (Radiomics, blue), clinical model (Clinics, green), and a combination of all models (Nomogram, Red). The y-axis measures the net benefit.

The decision curve analysis for both the radiomic model, clinical model, and the nomogram is presented in **Figure 5**. The decision curve showed that if the threshold probability is greater than 20%, using the nomogram in the current study to predict the recurrence time added more benefit than other forecast schemes or the forecast-none scheme. Within this range, net benefits were comparable on the basis of the radiomic nomogram and clinical model.

## Discussion

In this study, we found that radiomic features derived from preoperative brain MR images were associated with early recurrence in patients with GBM. Our predictive model combining radiomic features, VASARI parameters, and clinical variables could efficiently differentiate the patients with early recurrence from those with later recurrence. Thus, our study results demonstrate the potential usefulness of a non-invasive radiomic approach for predicting early recurrence of GBM.

Radiomics has been used to predict treatment response through assessment of tumor heterogeneity (32). Tumor heterogeneity, which includes variations in histological markers and in the presence of genetic alterations, has been associated with poor clinical outcomes in various diseases, including GBM (33). Heterogeneous tumors are more likely to contain cancer cells that may proliferate faster, may more readily metastasize, and may be more resistant to treatment (33, 34). Therefore, standard treatment with radiotherapy plus temozolomide may not be effective. At the same time, tumor heterogeneity often leads to vascular proliferation (33). A prior study showed that tumor vascularity is a prognostic factor for newly diagnosed glioblastoma (25). Higher tumor heterogeneity may reflect a more aggressive tumor and higher probability to recur (35). Therefore, it was not surprising that our radiomic model, which reflected tumor heterogeneity, achieved robust performance.

Our study found a renal function indicator, BUN, being relevant for predicting early recurrence in patients with GBM. BUN is a commonly used marker of renal function and its serum concentration varies according to glomerular filtration rate (32). High BUN indicates accumulation of ammonia in tissues and blood (33). In general, chemotherapy drugs such as cisplatin have strong renal toxicity and the dosage is determined and adjusted according to the BUN values. Therefore, abnormally elevated BUN may lead to a decrease in the dosage of chemotherapy drugs, which may result in inadequate treatment of the tumor and thus facilitate recurrence (34). In addition, elevated BUN is an indicator of poor physical condition of the patient, who may not be able to fight off tumor recurrence.

Our data showed that several VASARI parameters were independent factors for predicting early recurrence. The VASARI lexicon contains visually analyzable features extracted from routine medically indicated brain MRI and provides standardized visual grading of brain MRI findings for GBM. In our study, F5 and F21 were predictors of early recurrence, and F26 predicted the lack of early recurrence. The F5 parameter in the VASARI feature set indicates tumor enhancement, and a higher score means a higher enhancement ratio with more tumor blood supply and more enhancing tumor. Mathivet et al. (25) observed a progressive increase in vessel diameter during GBM development in an orthotopic mouse model. Therefore, it is reasonable to speculate that increased GBM tumor vascularity may support early recurrence. The VASARI parameter F21 indicates deep white matter invasion, which may pose challenges for complete tumor resection during surgery and render the tumor prone to recur. The VARASI parameter F26 indicates the extent of tumor resection when comparing the preoperative and postoperative MRI images. It is understandable that a more complete tumor resection should improve prognosis and made it less likely to recur.

This study had several limitations. First, this was a retrospective study at a single institution, limiting the generalizability of our study results. In addition, although there were 122 patients with GBM included in our study, our sample size was still modest for a radiomic study, given the heterogeneous nature of GBM. Second, we used 2D texture features of the brain MRI images. A 3D approach for textural features may offer more information about the entire tumor, which may improve predictive model performance (36). Lastly, we did not assess the overall survival rate for this cohort. Clinical data for some patients after recurrence were incomplete because they went back to their local hospitals to continue treatment. In addition, some patients were lost to long-term follow up. It has also been challenging to assess overall survival as patients with recurrent GBM underwent various therapies such as additional surgery, with or without concurrent chemoradiation, or radiation only or chemotherapy with temozolamide only or with addition of bevacizumab, immunotherapy, herbal holistic remedies, etc. With our modest sample size, we did not have the statistical power to tease out the survival rate for patients undergoing different treatments. A future multi-center prospective study of glioblastoma recurrence will be necessary to properly assess survival rate.

In summary, our MRI radiomic analysis and nomogram showed potential value for predicting early recurrence of GBM, which may assist in personalized treatment planning. Future prospective multicenter study with a larger sample size will be needed to validate our study result and to optimize the prediction models for clinical practice.

## Data Availability Statement

The original data will be made available to qualified researchers upon request.

## Ethics Statement

The studies involving human participants were reviewed and approved by Ethics Committee and Institutional Review Board of Xiangya Hospital of Central South University, P. R. China (IRB number: 201607831). Written informed consent for participation was not required for this study in accordance with the national legislation and the institutional requirements.

## Author Contributions

Conceived and designed the study: XY, JN, and ZT. Collected and analyzed the data: XY, YF, PP, HD, HT, ZDH, HL, GG, and ZLH. Prepared the manuscript: XY, JW, and BC. All authors contributed to the article and approved the submitted version.

## Funding

This study was funded in part by Natural Science Foundation of Hunan Province, P. R. China (2018JJ2641), and China Post-doctoral Science Foundation (2018M632997).

## Conflict of Interest

Author PP was employed by company GE Healthcare.

The remaining authors declare that the research was conducted in the absence of any commercial or financial relationships that could be construed as a potential conflict of interest.

## Publisher’s Note

All claims expressed in this article are solely those of the authors and do not necessarily represent those of their affiliated organizations, or those of the publisher, the editors and the reviewers. Any product that may be evaluated in this article, or claim that may be made by its manufacturer, is not guaranteed or endorsed by the publisher.
