# Supplementary material for: Preoperative Magnetic Resonance Imaging Radiomics for Predicting Early Recurrence of Glioblastoma
Source: Front Oncol. 2021 Oct 27;11:769188. doi: 10.3389/fonc.2021.769188 (PMC8579096; doi:10.3389/fonc.2021.769188)
Supplement: Supplementary file 1 [file DataSheet_1.docx]

# Supplementary Material

**Figure 1S.** Radiomic feature selection with the least absolute shrinkage and selection operator (LASSO) method.


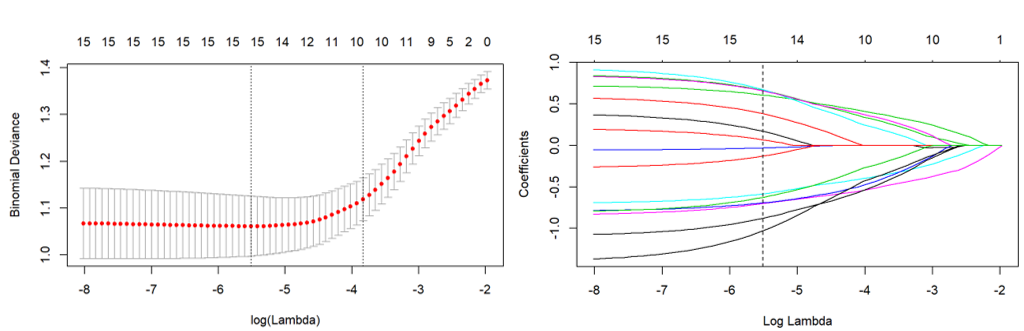


**Figure 2S.** Selection of the most predictive subset of features and evaluation of the corresponding coefficients.

**
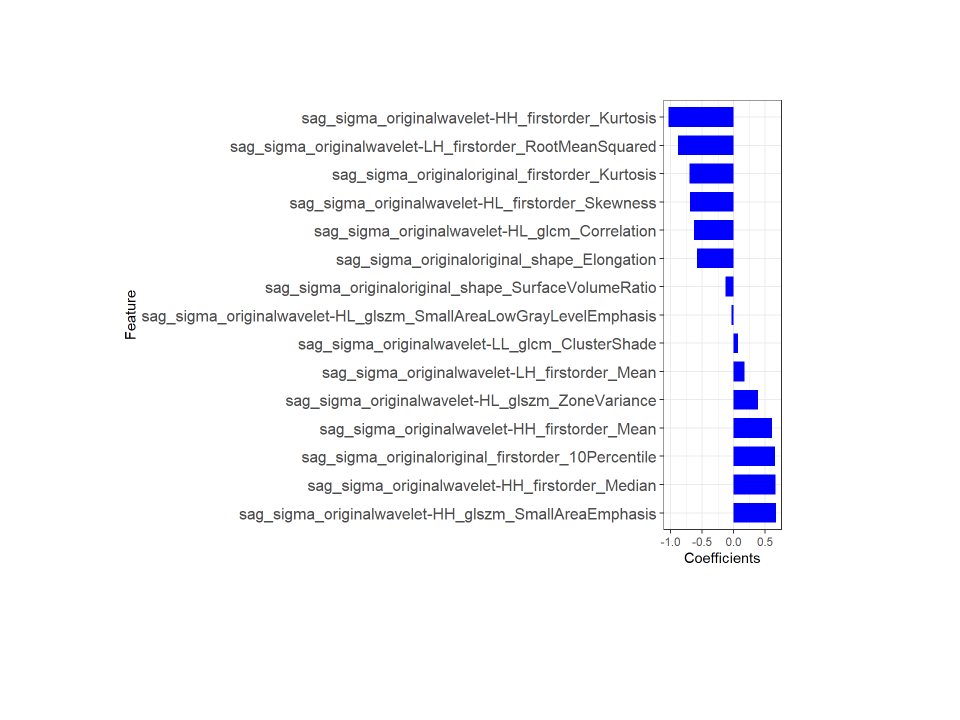
**
